# The Serial Engagement Model 17 Years After: From TCR Triggering to Immunotherapy

**DOI:** 10.3389/fimmu.2012.00272

**Published:** 2012-08-28

**Authors:** Salvatore Valitutti

**Affiliations:** ^1^INSERM, UMR 1043, Section Dynamique Moléculaire des Interactions Lymphocytaires, Centre de Physiopathologie de Toulouse PurpanToulouse, France; ^2^Université Toulouse III Paul-SabatierToulouse, France; ^3^Laboratoire d’Excellence Toulouse Cancer-TOUCANToulouse, France

**Keywords:** T cell antigen receptor, TCR serial engagement, T lymphocyte activation, signal transduction, immunological synapse

## Abstract

More than 15 years ago the serial engagement model was proposed as an attempt to solve the low affinity/high sensitivity paradox of TCR antigen recognition. Since then, the model has undergone ups and downs marked by the technical and conceptual advancements made in the field of T lymphocyte activation. Here, I describe the development of the model and survey recent literature providing evidence either for or against the idea that serial TCR/pMHC engagement might contribute to T lymphocyte activation. I also discuss how the concept of serial TCR engagement might be useful in the design of immunotherapeutic approaches aimed at potentiating T lymphocyte responses *in vivo*.

## Introduction

αβ T lymphocytes are activated by the engagement of their antigen receptors (TCR) with peptide/MHC complexes (pMHC) displayed on the surface of antigen presenting cells (APC). An apparent paradox in T lymphocyte biology is that the binding of a TCR with pMHC complexes displayed on the surface of APC has low affinity and a fast off-rate, yet allows the TCR to remain highly sensitive and specific to a particular antigen. How T cells decipher and amplify the information collected on the surface of APC and translate it into versatile biological responses is a central question in T cell biology.

The TCR serial engagement model was proposed as a stepping stone to address this unresolved question (Valitutti et al., 1995b). A drawback of this model is that it is not based on direct visualization of TCR dynamics at the T cell/APC contact site, but on an estimate of TCR/pMHC binding that uses TCR down-regulation as a parameter of TCR occupancy (Valitutti et al., 2010). As discussed below, this approach has been challenged. Moreover measurements of the TCR/pMHC binding parameters in solution provided results that were difficult to reconcile with the possibility that TCR might be rapidly and serially engaged by cell-bound pMHC (Stone et al., 2009). Collectively, these observations cast doubts on the idea that serial TCR engagement might actually contribute to the high level of sensitivity characteristic of T lymphocyte responses. Nevertheless, recent studies in which two-dimensional (2-D) binding parameters of TCR interaction with pMHC were considered, provided data in support of the idea that TCR/pMHC binding might be sequential, thus reopening the discussion on whether serial TCR engagement might take place at the T cell/APC contact site (Huang et al., 2010; He and Bongrand, 2012; Robert et al., 2012).

Here I discuss the development of the model and position the old serial engagement hypothesis in the context of what we currently know about T lymphocyte activation. Finally, I discuss how the concept of TCR serial engagement might be helpful in the design of therapies based on adoptive transfer of T cells carrying engineered tumor-specific TCR.

## The Serial TCR Engagement Model from an Historical Perspective

When the serial engagement model was proposed T lymphocyte activation was viewed as a paradox. Biochemical studies aimed at defining the minimum number of specific pMHC required to trigger T lymphocyte activation revealed that T cells could proliferate and produce cytokines in response to APC displaying as few as 50–100 specific pMHC complexes among a large number of structurally similar non-stimulatory pMHC (Demotz et al., 1990; Harding and Unanue, 1990). In addition, cytotoxic T cells (CTL) were reportedly able to kill target cells that were estimated to express as few as 1–10 specific pMHC (Sykulev et al., 1996). In spite of the high sensitivity of antigen recognition, the affinity of binding between TCR and pMHC appeared to be much lower than those measured for antibodies. Using different recombinant TCR and pMHC, the affinity of TCR/pMHC interaction in solution was estimated to range between 10−4 and 10−7 M with half-lives of seconds (Matsui et al., 1991, 1994; Corr et al., 1994). A complication to the high sensitivity/low affinity conundrum of T cell responses was the notion that T cell activation required sustained signaling, and needed to last for at least a few hours in order to promote cytokine secretion (Weiss et al., 1987; Goldsmith and Weiss, 1988; Gray et al., 1988).

During the last 15 years, the development of high-resolution imaging techniques moved the focus of investigation from the measurement of functional and phenotypic parameters in cell populations to the visualization of molecular dynamics in individual cells at the level of the immunological synapse (IS, Monks et al., 1998; Grakoui et al., 1999). Recent studies clearly illustrate the high sensitivity of individual T cells to antigenic stimulation, as well as the importance of sustained signaling for T cell activation. In one such study, work by M.M. Davis and colleagues, showed that murine CD4^+^ T cells undergo sustained [Ca^2+^]_i_ increase when interacting with APC that display as few as 10–15 specific pMHC at the IS, demonstrating that sustained signaling is actually triggered by a few antigen specific ligands (Irvine et al., 2002). Sustained activation of signaling pathways in T cells (including Ca^2+^-calcineurin pathway, PI3K pathway, and PKCθ pathway, etc.) has been mechanistically linked to the nuclear translocation of transcription factors required for cytokine production and proliferation (Timmerman et al., 1996; Fabre et al., 2005; Zanin-Zhorov et al., 2011). Moreover, it has also been shown that, not only the duration of signaling, but also the intensity and the frequency of signal oscillations are important factors that modulate the outcome of T cell responses (Dolmetsch et al., 1998; Utzny et al., 2005).

About 17 years ago, we observed that when TCR/pMHC interactions in pre-formed conjugates of T cells and APC were blocked by the addition of anti-MHC Class II antibodies, [Ca^2+^]_i_ increases ceased within a few minutes and T cells failed to produce IFN-γ (Valitutti et al., 1995a). This finding suggested that sustained signaling resulted from the prolonged and uninterrupted engagement of TCR with pMHC displayed on the APC surface. A functional actin cytoskeleton appeared to be instrumental for this process by allowing T cells to scan the APC surface and to form areas of tight adhesion with the opposing cell membrane (Valitutti et al., 1995a). These observations, particularly evident at low antigenic densities, raised the question of how TCR could remain bound to the same few pMHC for long enough to achieve sustained signaling within dynamic T cell/APC contacts.

To determine the number of TCR triggered by pMHC, we measured TCR down-regulation (i.e., the reduction of TCR expression on the cell surface due to internalization and targeting to lysosomes (Valitutti et al., 1997), and compared it to immuno-precipitation of iodinated peptides bound to MHC molecules. We calculated that, in T cells interacting with APC displaying low antigenic pMHC densities (∼100 pMHC per APC), a large number of TCR were triggered (up to 18,000 per T cell; Valitutti et al., 1995b). The above estimates, together with the observation that sustained signaling in T cells required uninterrupted TCR engagement inspired the serial engagement model (Valitutti et al., 1995b; Valitutti and Lanzavecchia, 1997).

**Figure 1:**
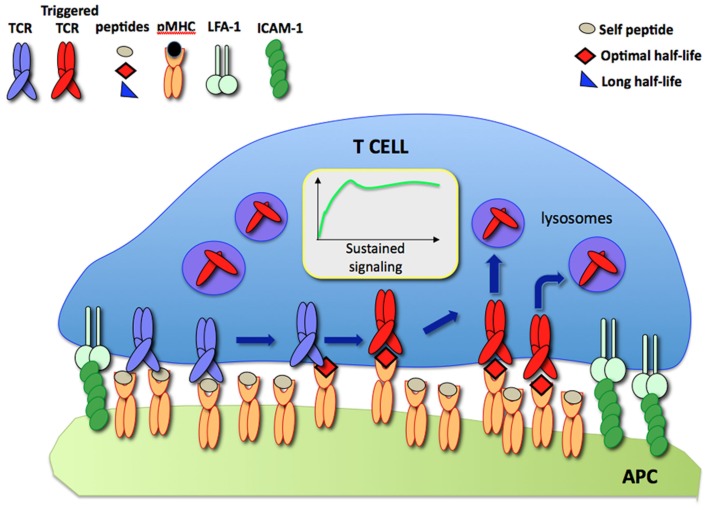
**The serial TCR engagement model**. At the IS, a few specific pMHC (red) sequentially trigger incoming TCR resulting in sustained signaling. Triggered TCR are internalized and targeted to lysosomes for degradation while unbound pMHC bind new TCR.

According to the model, during a dynamic T cell/APC interaction an increasing number of TCR are engaged and triggered by a small number of specific pMHC displayed on the APC surface resulting in sustained signaling. Such a process is compatible with the short half-lives of TCR/pMHC binding which have been observed in other studies (Figure 1).

The use of TCR down-regulation as a measure of TCR occupancy has been debated. It has been reported that in dual-TCR-expressing T cells a substantial fraction of non-engaged TCR could be co-modulated in a bystander fashion (San Jose et al., 2000). As discussed elsewhere (Valitutti et al., 2010), the various studies addressing bystander TCR co-modulation are contradictory. Moreover, the extent to which co-modulation is observed appears to be related to the type of cells used in the different studies. For example, co-modulation is often reported as a phenomenon in Jurkat T cells, but is rarely seen (or reported to be limited) in studies with human T cell clones or mouse primary cells (Valitutti et al., 1995b; Niedergang et al., 1997; San Jose et al., 2000; Schrum and Turka, 2002; Bonefeld et al., 2003; Gladow et al., 2004). Why differences among cell systems exist is presently unclear. It is likely that the extent of detectable TCR co-modulation may depend on various cellular parameters, including the fraction of monomeric versus pre-aggregated TCR present on the surface of different T cells. This issue is still debated. One study reported that TCR/CD3 complexes are expressed on the T cell surface essentially as monomers (James et al., 2007), while others reported that TCR are, in part, pre-aggregated forming nano-clusters (Schamel et al., 2005; Lillemeier et al., 2010). Discrepancies among these studies might be due to the different experimental approaches used to determine TCR clustering. Further research is required to address this key point.

In conclusion, in its original formulation the serial engagement model offered a plausible explanation to the low affinity/high sensitivity paradox of T cell activation and suggested a mechanism for the induction of sustained signaling by a few antigenic ligands. However, the original idea of comparing the number of pMHC with the number of down-regulated TCR is *per se* a limitation of the model. Serial TCR engagement needs to be further assessed with modern and more direct experimental approaches.

## 3-D Versus 2-D TCR/pMHC Interactions

**Figure 2:**
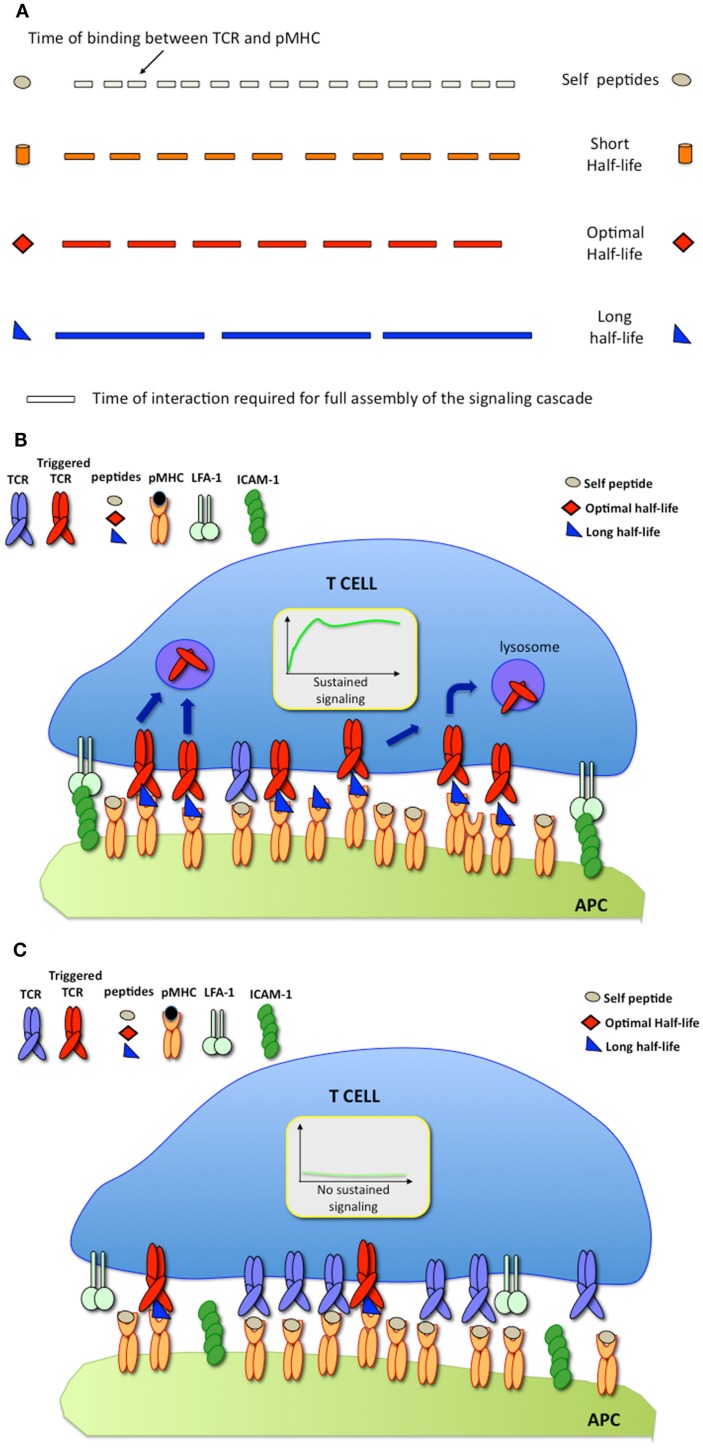
**(A)** The serial engagement model postulates that pMHC ligands exhibiting optimal binding half-lives to TCR behave as optimal agonists; **(B)** At high pMHC densities, pMHC exhibiting long binding half-lives are stimulatory; **(C)** At low pMHC densities, pMHC exhibiting long binding half-lives fail to trigger T cell responses.

The serial engagement model predicts that there is a defined window of half-lives of TCR-pMHC binding required for optimal T cell activation (Valitutti and Lanzavecchia, 1997). While short half-lives (with rapid TCR/pMHC binding off-rates) prevent productive TCR triggering, as stated by the kinetics-proofreading model (Rabinowitz et al., 1996), long half-lives (with slow binding off-rates) reduce the efficiency of TCR serial engagement (Figure 2A). However, measurements of TCR/pMHC binding parameters in solution (defined as three-dimensional or 3-D parameters) using surface plasmon resonance (SPR) or comparing the binding of different pMHC tetramers to T cells, provided results that were inconsistent with the optimal half-life hypothesis. While this hypothesis is supported by computational studies (Wofsy et al., 2001; Coombs et al., 2002) and by some experimental results (Hudrisier et al., 1998; Kalergis et al., 2001; Cemerski et al., 2007; Adams et al., 2011), other studies failed to provide evidence for an optimal half-life window (Holler et al., 2001; Tian et al., 2007). It is possible that some of the discrepancies arise from the different readouts used to monitor T cell activation in the different studies (Corse et al., 2011). Recent work has been able to reconcile these apparently contrasting results by showing that, depending on the on-rate of binding, the potency of some pMHC ligands for stimulating T cells correlates better with pMHC/TCR affinity, while the stimulation potency of others is determined instead by an optimal half-life (Aleksic et al., 2010; Govern et al., 2010). It is important to note that, SPR and tetramer measurements, although useful to compare different TCR ligands, rely on parameters that are estimated from 3-D binding, a condition that might not accurately mimic the situation within the confines of a T cell/APC IS.

Recent technological breakthroughs have made it possible to measure TCR/pMHC binding using experimental approaches that more accurately mimic the 2-D TCR/pMHC interaction within the IS (He and Bongrand, 2012).

Huppa et al. investigated the interaction between TCR labeled with fluorescent antibody and expressed on the cell surface, with pMHC embedded in supported lipid bilayers using fluorescence resonance energy transfer (FRET). This analysis showed that in 2-D the on-rate of binding between TCR and pMHC is much faster than that measured in 3-D, and that the dissociation rate of TCR/pMHC bonds in 2-D is 4- to 12-fold more rapid than the rate measured in 3-D. Interestingly, when T cell actin cytoskeleton is poisoned, the differences between 3-D and 2-D off-rates are abolished, indicating that the accelerated off-rates observed in 2-D depend on active cellular dynamics. The study also showed that the 2-D binding affinity is high and correlates with ligand potency, in agreement with previous SPR measurements (Huppa et al., 2010).

Huang at al. used micropipettes to immobilize T cells and to form contacts with either red blood cells or beads coated with pMHC to estimated the 2-D on/off-rates of binding by monitoring either red blood cell deformation or thermal fluctuation in the TCR/pMHC binding. This approach reports very fast 2-D on-rates. Surprisingly, off-rates of binding were also found to be extremely rapid (∼8,000 times faster than those measured in solution) and are faster for agonist pMHC ligands than for weak ligands, suggesting serial TCR engagement (Huang et al., 2010). The importance of considering both 3-D and 2-D binding kinetics is emphasized by a recent study by K. C. Garcia and colleagues. They show that TCR/pMHC binding parameters are different if measured in solution (in the absence of physical constraints) or in 2-D (Adams et al., 2011).

Finally, in a recent and detailed study, Robert et al. used TCR coated microbeads in laminar flow chambers and measured their interaction with surface-immobilized pMHC under cell free conditions (Pierres et al., 1996; Robert et al., 2012). This analysis showed that 2-D dissociation rates are comparable to 3-D dissociation rates when measured in a cell free system such as this, supporting the idea that accelerated 2-D dissociations of TCR/pMHC bonds found in other studies (Huang et al., 2010; Huppa et al., 2010), result from active cellular dynamics. Robert et al. (2012) also found that T cell activation increases with a longer binding half-life, but negatively correlates with bond mechanical strength. As discussed below, these results reconcile SPR measurements (reporting that ligand potency increases with binding half-life, Holler et al., 2001; Tian et al., 2007) with the requirement of rapid bond disruption and re-formation for serial TCR triggering (Valitutti and Lanzavecchia, 1997). These observations are also compatible with recent studies showing that TCR can function as mechanosensors (e.g., receptors converting mechanical energy into biochemical signals; Kim et al., 2009, 2012; Li et al., 2010; Husson et al., 2011; He and Bongrand, 2012) and with models of T cell activation postulating that mechanical forces are involved in TCR triggering (Ma and Finkel, 2010).

Based on these findings, a current view of T cell activation posits that cytoskeletal movements can fine-tune the half-life of TCR/pMHC interactions in live T cell/APC conjugates. Optimal TCR ligands would have long enough half-lives to trigger receptors but low mechanical strength. Together, these parameters would allow TCR/pMHC bonds to rapidly dissociate and reform within dynamic T cell/APC contacts thus enhancing serial TCR triggering (Ma and Finkel, 2010; Robert et al., 2012). It is interesting to note that this view goes back to initial observations showing that a functional actin cytoskeleton and T cell motility are instrumental for sustained signaling in T cells triggered by cell-bound ligands (Valitutti et al., 1995a).

In conclusion, results obtained from 2-D measurements are still too limited and somewhat contradictory to either support or refute the model of serial TCR engagement. Further work, using additional panels of TCR/pMHC pairs and well-standardized 2-D assays, is required to convincingly test the model. However, by showing that binding off-rates can be faster at cellular interfaces due to cytoskeleton-driven dynamics and that bond mechanical strength might influences ligand potency, 2-D methods have re-opened the discussion on the purported relevance of serial TCR engagement.

## Could TCR Serial Engagement Be Relevant for Immunotherapy?

### The importance of ligand density

Since its inception, it was implicit that the model of TCR serial engagement would only apply under conditions in which T cells interacted with APC displaying a very small number of antigenic ligands. At high densities of cognate pMHC, TCR serial engagement would be not only difficult to envisage, but also unnecessary. Thus, optimal half-lives of TCR/pMHC binding would be required for serial TCR engagement only at low antigen densities (Figures 2B,C).

The first direct evidence supporting this idea came from a study by Gonzales et al. in which the relationship between the density of antigenic ligands and their affinity for TCR was investigated. This study provides computational and experimental data showing that an appropriate TCR/pMHC half-life window is only required to elicit T cell responses at low pMHC densities (Gonzalez et al., 2005). Furthermore, Dushek et al. recently emphasized the importance of the strength of antigenic stimulation when comparing “affinity” versus “productive hit rate” models of T cell activation. They employed computational and experimental approaches to correlate solution measurements of TCR/pMHC binding parameters with the potency of a panel of pMHC variants. They reported that dose-responses are better indicators of T cell activation than EC_50_ (half-maximal effective concentration) when relating TCR/pMHC binding affinities to elicited responses (Dushek et al., 2011). Direct evidence that serial TCR engagement is dispensable in conditions in which a strong stimulation is provided to T cells also comes from recent findings showing that, when a substantial fraction of TCR is cross-linked with monovalent pMHC embedded in supported lipid bilayers, T cell activation is sustained and amplified (Xie et al., 2012).

### Requirement for an optimal affinity window in clinically oriented studies

Understanding whether or not TCR exhibiting an intermediate affinity might efficiently trigger T cell activation at low antigen densities, is relevant for cancer immunotherapies that are based on adoptive T cell transfer. In contrast to infectious diseases, malignant neoplastic diseases are characterized as having relatively slow progression and displaying low antigen densities. A study in which the functional properties of T cells transduced with TCR exhibiting various binding affinities were compared *in vitro*, reported that human T cells transduced with TCR of intermediate affinity are sensitive to low antigenic stimuli and do not exhibit cross-reactivity (Zhao et al., 2007). Recent clinically oriented studies further illustrate the importance of the balance between the strength of antigenic stimulation and TCR binding properties. Thomas et al. showed that human non-transformed T cells transduced with affinity-matured TCR (specific for a HIV-derived Gag peptide) that exhibit 700-fold increased affinity to its cognate pMHC, require much higher pMHC densities to trigger T cell responses than wild-type TCR (Thomas et al., 2011). Irving et al. used a rational design to generate a panel of human TCR specific for a tumor antigen peptide and that exhibited a variety of affinity/off-rates (measured in 3-D using SPR). They show that human non-transformed T cells transduced with TCR engineered to have supraphysiologic affinity for pMHC, exhibit defective responses at low/intermediate antigen doses. By increasing antigen dose, the deficiency of high affinity TCR to elicit optimal T cell responses is gradually overcome (Irving et al., 2012).

In conclusion, increasing evidence indicates that appropriate TCR/pMHC binding half-lives would, at low antigen densities, potentiate T cell responses. However, on the basis of available data, it is difficult to predict whether TCR ligands with an intermediate affinity might elicit stronger responses *in vivo* and, in turn, substantially improve the outcome of immunotherapies based on T lymphocyte adoptive transfer. It may be important to test this hypothesis in clinics and therefore consider T cell-based therapies using intermediate affinity engineered TCR.

## Concluding Remarks

More than 17 years after its proposal the serial TCR engagement model is still avidly debated. Recent observations showing that TCR with intermediate binding half-lives for pMHC can efficiently trigger T cells at low antigen densities, and that TCR/pMHC dissociation rates in 2-D are very rapid suggest that serial TCR engagement events might actually occur within the confines of T cell/APC synapses. However, available data are contradictory. It is unlikely that the serial TCR engagement model will be able to explain results obtained in all the different systems and experimental conditions. Several parameters, such as the strength and quality of antigenic stimuli, the quality of the APC and the context in which antigen presentation takes place, influence T cell responses. The fact that available data have been obtained using different T cell models (such T cell hybridomas, transgenic T cell systems, non-transformed T cells, etc.) does not help clarify our understanding on whether or not, and to what extent, TCR serial engagement might contribute to T cell activation.

In the future it should be possible to address these unresolved issues. For example, the recent development of high-resolution live cell microscopy techniques should allow the visualization of the dynamics of individual TCR/pMHC encounters at the IS by combining single particle tracking with other florescence microscopy techniques. These approaches will allow investigators to “see” under which conditions serial engagement may take place. Furthermore, the necessity of testing panels of T cells carrying engineered tumor-specific TCR for adoptive transfer therapies, will allow systematic and in-depth analysis of 3-D and 2-D TCR/pMHC binding parameters and help to clarify the relationship between different binding parameters and stimulation potency.

It is intriguing that these clinically oriented studies are bringing back the idea of serial TCR engagement to the experimental system in which it was originally conceived: human non-transformed T cells. The actual relevance of serial TCR engagement in the process of T cell activation in different T cell systems is still elusive and may well be limited. Nevertheless, it would be an excellent outcome for this model if it can contribute, at least to a certain extent, to the design of immunological strategies to fight cancer.

## Conflict of Interest Statement

The author declares that the research was conducted in the absence of any commercial or financial relationships that could be construed as a potential conflict of interest.

## References

[B1] Adams J. J. Narayanan S. Liu B. Birnbaum M. E. Kruse A. C. Bowerman N. A. Chen W. Levin A. M. Connolly J. M. Zhu C. Kranz D. M. Garcia K. C. (2011). T cell receptor signaling is limited by docking geometry to peptide-major histocompatibility complex. Immunity 35, 681–693 10.1016/j.immuni.2011.09.013 22101157 PMC3253265

[B2] Aleksic M. Dushek O. Zhang H. Shenderov E. Chen J. L. Cerundolo V. Coombs D. Van Der Merwe P. A. (2010). Dependence of T cell antigen recognition on T cell receptor-peptide MHC confinement time. Immunity 32, 163–174 10.1016/j.immuni.2009.11.013 20137987 PMC2862301

[B3] Bonefeld C. M. Rasmussen A. B. Lauritsen J. P. Von Essen M. Odum N. Andersen P. S. Geisler C. (2003). TCR comodulation of nonengaged TCR takes place by a protein kinase C and CD3 gamma di-leucine-based motif-dependent mechanism. J. Immunol. 171, 3003–3009 12960325 10.4049/jimmunol.171.6.3003

[B4] Cemerski S. Das J. Locasale J. Arnold P. Giurisato E. Markiewicz M. A. Fremont D. Allen P. M. Chakraborty A. K. Shaw A. S. (2007). The stimulatory potency of T cell antigens is influenced by the formation of the immunological synapse. Immunity 26, 345–355 10.1016/j.immuni.2007.01.013 17346997 PMC2763191

[B5] Coombs D. Kalergis A. M. Nathenson S. G. Wofsy C. Goldstein B. (2002). Activated TCRs remain marked for internalization after dissociation from pMHC. Nat. Immunol. 3, 926–931 10.1038/ni838 12244312

[B6] Corr M. Slanetz A. E. Boyd L. F. Jelonek M. T. Khilko S. Al-Ramadi B. K. Kim Y. S. Maher S. E. Bothwell A. L. Margulies D. H. (1994). T cell receptor-MHC class I peptide interactions: affinity, kinetics, and specificity. Science 265, 946–949 10.1126/science.8052850 8052850

[B7] Corse E. Gottschalk R. A. Allison J. P. (2011). Strength of TCR-peptide/MHC interactions and in vivo T cell responses. J. Immunol. 186, 5039–5045 10.4049/jimmunol.1003650 21505216

[B8] Demotz S. Grey H. M. Sette A. (1990). The minimal number of class II MHC-antigen complexes needed for T cell activation. Science 249, 1028–1030 10.1126/science.2118680 2118680

[B9] Dolmetsch R. E. Xu K. Lewis R. S. (1998). Calcium oscillations increase the efficiency and specificity of gene expression. Nature 392, 933–936 10.1038/31960 9582075

[B10] Dushek O. Aleksic M. Wheeler R. J. Zhang H. Cordoba S. P. Peng Y. C. Chen J. L. Cerundolo V. Dong T. Coombs D. Van Der Merwe P. A. (2011). Antigen potency and maximal efficacy reveal a mechanism of efficient T cell activation. Sci. Signal. 4, ra39 10.1126/scisignal.2001430 21653229 PMC4143974

[B11] Fabre S. Lang V. Harriague J. Jobart A. Unterman T. G. Trautmann A. Bismuth G. (2005). Stable activation of phosphatidylinositol 3-kinase in the T cell immunological synapse stimulates Akt signaling to FoxO1 nuclear exclusion and cell growth control. J. Immunol. 174, 4161–4171 15778376 10.4049/jimmunol.174.7.4161

[B12] Gladow M. Uckert W. Blankenstein T. (2004). Dual T cell receptor T cells with two defined specificities mediate tumor suppression via both receptors. Eur. J. Immunol. 34, 1882–1891 10.1002/eji.200425041 15214036

[B13] Goldsmith M. A. Weiss A. (1988). Early signal transduction by the antigen receptor without commitment to T cell activation. Science 240, 1029–1031 10.1126/science.3259335 3259335

[B14] Gonzalez P. A. Carreno L. J. Coombs D. Mora J. E. Palmieri E. Goldstein B. Nathenson S. G. Kalergis A. M. (2005). T cell receptor binding kinetics required for T cell activation depend on the density of cognate ligand on the antigen-presenting cell. Proc. Natl. Acad. Sci. U.S.A. 102, 4824–4829 10.1073/pnas.0409517102 15772168 PMC555720

[B15] Govern C. C. Paczosa M. K. Chakraborty A. K. Huseby E. S. (2010). Fast on-rates allow short dwell time ligands to activate T cells. Proc. Natl. Acad. Sci. U.S.A. 107, 8724–8729 10.1073/pnas.1000966107 20421471 PMC2889346

[B16] Grakoui A. Bromley S. K. Sumen C. Davis M. M. Shaw A. S. Allen P. M. Dustin M. L. (1999). The immunological synapse: a molecular machine controlling T cell activation. Science 285, 221–227 10.1126/science.285.5425.221 10398592

[B17] Gray L. S. Gnarra J. R. Sullivan J. A. Mandell G. L. Engelhard V. H. (1988). Spatial and temporal characteristics of the increase in intracellular Ca2+ induced in cytotoxic T lymphocytes by cellular antigen. J. Immunol. 141, 2424–2430 3262660

[B18] Harding C. V. Unanue E. R. (1990). Quantitation of antigen-presenting cell MHC class II/peptide complexes necessary for T-cell stimulation. Nature 346, 574–576 10.1038/346574a0 2115981

[B19] He H. T. Bongrand P. (2012). Membrane dynamics shape TCR-generated signaling. Front. Immunol. 3, 90 10.3389/fimmu.2012.00090 22566969 PMC3342369

[B20] Holler P. D. Lim A. R. Cho B. K. Rund L. A. Kranz D. M. (2001). CD8(−) T cell transfectants that express a high affinity T cell receptor exhibit enhanced peptide-dependent activation. J. Exp. Med. 194, 1043–1052 10.1084/jem.194.8.1043 11602635 PMC2193521

[B21] Huang J. Zarnitsyna V. I. Liu B. Edwards L. J. Jiang N. Evavold B. D. Zhu C. (2010). The kinetics of two-dimensional TCR and pMHC interactions determine T-cell responsiveness. Nature 464, 932–936 10.1038/nature08944 20357766 PMC2925443

[B22] Hudrisier D. Kessler B. Valitutti S. Horvath C. Cerottini J. C. Luescher I. F. (1998). The efficiency of antigen recognition by CD8+ CTL clones is determined by the frequency of serial TCR engagement. J. Immunol. 161, 553–562 9670927

[B23] Huppa J. B. Axmann M. Mortelmaier M. A. Lillemeier B. F. Newell E. W. Brameshuber M. Klein L. O. Schutz G. J. Davis M. M. (2010). TCR-peptide-MHC interactions in situ show accelerated kinetics and increased affinity. Nature 463, 963–967 10.1038/nature08746 20164930 PMC3273423

[B24] Husson J. Chemin K. Bohineust A. Hivroz C. Henry N. (2011). Force generation upon T cell receptor engagement. PLoS ONE 6, e19680 10.1371/journal.pone.0019680 21572959 PMC3091878

[B25] Irvine D. J. Purbhoo M. A. Krogsgaard M. Davis M. M. (2002). Direct observation of ligand recognition by T cells. Nature 419, 845–849 10.1038/nature01076 12397360

[B26] Irving M. Zoete V. Hebeisen M. Schmid D. Baumgartner P. Guillaume P. Romero P. Speiser D. Luescher I. Rufer N. Michielin O. (2012). Interplay between T cell receptor binding kinetics and the level of cognate peptide presented by major histocompatibility complexes governs CD8+ T cell responsiveness. J. Biol. Chem. 287, 23068–23078 22549784 10.1074/jbc.M112.357673 PMC3391157

[B27] James J. R. White S. S. Clarke R. W. Johansen A. M. Dunne P. D. Sleep D. L. Fitzgerald W. J. Davis S. J. Klenerman D. (2007). Single-molecule level analysis of the subunit composition of the T cell receptor on live T cells. Proc. Natl. Acad. Sci. U.S.A. 104, 17662–17667 10.1073/pnas.0609174104 17971442 PMC2077052

[B28] Kalergis A. M. Boucheron N. Doucey M. A. Palmieri E. Goyarts E. C. Vegh Z. Luescher I. F. Nathenson S. G. (2001). Efficient T cell activation requires an optimal dwell-time of interaction between the TCR and the pMHC complex. Nat. Immunol. 2, 229–234 10.1038/35067042 11224522

[B29] Kim S. T. Shin Y. Brazin K. Mallis R. J. Sun Z. Y. Wagner G. Lang M. J. Reinherz E. L. (2012). TCR Mechanobiology: torques and tunable structures linked to early T cell signaling. Front. Immunol. 3, 76 10.3389/fimmu.2012.00076 22566957 PMC3342345

[B30] Kim S. T. Takeuchi K. Sun Z. Y. Touma M. Castro C. E. Fahmy A. Lang M. J. Wagner G. Reinherz E. L. (2009). The alphabeta T cell receptor is an anisotropic mechanosensor. J. Biol. Chem. 284, 31028–31037 10.1074/jbc.M109.000752 19755427 PMC2781503

[B31] Li Y. C. Chen B. M. Wu P. C. Cheng T. L. Kao L. S. Tao M. H. Lieber A. Roffler S. R. (2010). Cutting Edge: mechanical forces acting on T cells immobilized via the TCR complex can trigger TCR signaling. J. Immunol. 184, 5959–5963 10.4049/jimmunol.0900775 20435924

[B32] Lillemeier B. F. Mortelmaier M. A. Forstner M. B. Huppa J. B. Groves J. T. Davis M. M. (2010). TCR and Lat are expressed on separate protein islands on T cell membranes and concatenate during activation. Nat. Immunol. 11, 90–96 10.1038/nrm2842 20010844 PMC3273422

[B33] Ma Z. Finkel T. H. (2010). T cell receptor triggering by force. Trends Immunol. 31, 1–6 10.1016/j.it.2009.09.008 19836999 PMC2818226

[B34] Matsui K. Boniface J. J. Reay P. A. Schild H. Fazekas De St Groth B. Davis M. M. (1991). Low affinity interaction of peptide-MHC complexes with T cell receptors. Science 254, 1788–1791 10.1126/science.1763329 1763329

[B35] Matsui K. Boniface J. J. Steffner P. Reay P. A. Davis M. M. (1994). Kinetics of T-cell receptor binding to peptide/I-Ek complexes: correlation of the dissociation rate with T-cell responsiveness. Proc. Natl. Acad. Sci. U.S.A. 91, 12862–12866 10.1073/pnas.91.26.12862 7809136 PMC45540

[B36] Monks C. R. Freiberg B. A. Kupfer H. Sciaky N. Kupfer A. (1998). Three-dimensional segregation of supramolecular activation clusters in T cells. Nature 395, 82–86 10.1038/25764 9738502

[B37] Niedergang F. Dautry-Varsat A. Alcover A. (1997). Peptide antigen or superantigen-induced down-regulation of TCRs involves both stimulated and unstimulated receptors. J. Immunol. 159, 1703–1710 9257831

[B38] Pierres A. Benoliel A. M. Bongrand P. (1996). Measuring bonds between surface-associated molecules. J. Immunol. Methods 196, 105–120 10.1016/0022-1759(96)00103-2 8841450

[B39] Rabinowitz J. D. Beeson C. Lyons D. S. Davis M. M. Mcconnell H. M. (1996). Kinetic discrimination in T-cell activation. Proc. Natl. Acad. Sci. U.S.A. 93, 1401–1405 10.1073/pnas.93.13.6421 8643643 PMC39950

[B40] Robert P. Aleksic M. Dushek O. Cerundolo V. Bongrand P. Van Der Merwe P. A. (2012). Kinetics and mechanics of two-dimensional interactions between T cell receptors and different activating ligands. Biophys. J. 102, 248–257 10.1016/j.bpj.2011.11.1367 22339861 PMC3260781

[B41] San Jose E. Borroto A. Niedergang F. Alcover A. Alarcon B. (2000). Triggering the TCR complex causes the downregulation of nonengaged receptors by a signal transduction-dependent mechanism. Immunity 12, 161–170 10.1016/S1074-7613(00)80169-7 10714682

[B42] Schamel W. W. Arechaga I. Risueno R. M. Van Santen H. M. Cabezas P. Risco C. Valpuesta J. M. Alarcon B. (2005). Coexistence of multivalent and monovalent TCRs explains high sensitivity and wide range of response. J. Exp. Med. 202, 493–503 10.1084/jem.20042155 16087711 PMC2212847

[B43] Schrum A. G. Turka L. A. (2002). The proliferative capacity of individual naive CD4(+) T cells is amplified by prolonged T cell antigen receptor triggering. J. Exp. Med. 196, 793–803 10.1084/jem.20020158 12235212 PMC2194051

[B44] Stone J. D. Chervin A. S. Kranz D. M. (2009). T-cell receptor binding affinities and kinetics: impact on T-cell activity and specificity. Immunology 126, 165–176 10.1111/j.1365-2567.2008.03015.x 19125887 PMC2632691

[B45] Sykulev Y. Joo M. Vturina I. Tsomides T. J. Eisen H. N. (1996). Evidence that a single peptide-MHC complex on a target cell can elicit a cytolytic T cell response. Immunity 4, 565–571 10.1016/S1074-7613(00)80483-5 8673703

[B46] Thomas S. Xue S. A. Bangham C. R. Jakobsen B. K. Morris E. C. Stauss H. J. (2011). Human T cells expressing affinity-matured TCR display accelerated responses but fail to recognize low density of MHC-peptide antigen. Blood 118, 319–329 10.1182/blood-2011-04-349258 21606483

[B47] Tian S. Maile R. Collins E. J. Frelinger J. A. (2007). CD8+ T Cell activation is governed by TCR-peptide/MHC affinity, not dissociation rate. J. Immunol. 179, 2952–2960 17709510 10.4049/jimmunol.179.5.2952

[B48] Timmerman L. A. Clipstone N. A. Ho S. N. Northrop J. P. Crabtree G. R. (1996). Rapid shuttling of NF-AT in discrimination of Ca2+ signals and immunosuppression. Nature 383, 837–840 10.1038/383837a0 8893011

[B49] Utzny C. Faroudi M. Valitutti S. (2005). Frequency encoding of T-cell receptor engagement dynamics in calcium time series. Biophys. J. 88, 1–14 10.1529/biophysj.104.053884 15501938 PMC1304989

[B50] Valitutti S. Coombs D. Dupre L. (2010). The space and time frames of T cell activation at the immunological synapse. FEBS Lett. 584, 4851–4857 10.1016/j.febslet.2010.10.010 20940018

[B51] Valitutti S. Dessing M. Aktories K. Gallati H. Lanzavecchia A. (1995a). Sustained signaling leading to T cell activation results from prolonged T cell receptor occupancy. Role of T cell actin cytoskeleton. J. Exp. Med. 181, 577–584 10.1084/jem.181.2.577 7836913 PMC2191861

[B52] Valitutti S. Muller S. Cella M. Padovan E. Lanzavecchia A. (1995b). Serial triggering of many T-cell receptors by a few peptide-MHC complexes. Nature 375, 148–151 10.1038/375148a0 7753171

[B53] Valitutti S. Lanzavecchia A. (1997). Serial triggering of TCRs: a basis for the sensitivity and specificity of antigen recognition. Immunol. Today 18, 299–304 10.1016/S0167-5699(97)80027-8 9190117

[B54] Valitutti S. Muller S. Salio M. Lanzavecchia A. (1997). Degradation of T cell receptor (TCR)-CD3-zeta complexes after antigenic stimulation. J. Exp. Med. 185, 1859–1864 10.1084/jem.185.10.1859 9151711 PMC2196323

[B55] Weiss A. Shields R. Newton M. Manger B. Imboden J. (1987). Ligand-receptor interactions required for commitment to the activation of the interleukin 2 gene. J. Immunol. 138, 2169–2176 3104454

[B56] Wofsy C. Coombs D. Goldstein B. (2001). Calculations show substantial serial engagement of T cell receptors. Biophys. J. 80, 606–612 10.1016/S0006-3495(01)76041-9 11159429 PMC1301260

[B57] Xie J. Huppa J. B. Newell E. W. Huang J. Ebert P. J. Li Q. J. Davis M. M. (2012). Photocrosslinkable pMHC monomers stain T cells specifically and cause ligand-bound TCRs to be “preferentially” transported to the cSMAC. Nat. Immunol. 13, 674–680 10.1038/ni.2344 22660579 PMC3645478

[B58] Zanin-Zhorov A. Dustin M. L. Blazar B. R. (2011). PKC-theta function at the immunological synapse: prospects for therapeutic targeting. Trends Immunol. 32, 358–363 10.1016/j.it.2011.04.007 21733754 PMC3573858

[B59] Zhao Y. Bennett A. D. Zheng Z. Wang Q. J. Robbins P. F. Yu L. Y. Li Y. Molloy P. E. Dunn S. M. Jakobsen B. K. Rosenberg S. A. Morgan R. A. (2007). High-affinity TCRs generated by phage display provide CD4+ T cells with the ability to recognize and kill tumor cell lines. J. Immunol. 179, 5845–5854 17947658 10.4049/jimmunol.179.9.5845 PMC2140228

